# Altered Coupling Between Resting-State Cerebral Blood Flow and Functional Connectivity Strength in Cervical Spondylotic Myelopathy Patients

**DOI:** 10.3389/fneur.2021.713520

**Published:** 2021-09-08

**Authors:** Wuzeng Wei, Tao Wang, Tuersong Abulizi, Bing Li, Jun Liu

**Affiliations:** ^1^Department of Joints, Tianjin Hospital, Tianjin University, Tianjin, China; ^2^Clinical College of Orthopedics, Tianjin Medical University, Tianjin, China

**Keywords:** arterial spin labeling, cerebral blood flow, functional magnetic resonance imaging, functional connectivity, cervical spondylotic myelopathy, resting-state fMRI

## Abstract

**Background:** Changes in regional neural activity and functional connectivity in cervical spondylotic myelopathy (CSM) patients have been reported. However, resting-state cerebral blood flow (CBF) changes and coupling between CBF and functional connectivity in CSM patients are largely unknown.

**Methods:** Twenty-seven CSM patients and 24 sex/age-matched healthy participants underwent resting-state functional MRI and arterial spin labeling imaging to compare functional connectivity strength (FCS) and CBF between the two groups. The CBF–FCS coupling of the whole gray matter and specific regions of interest was also compared between the groups.

**Results:** Compared with healthy individuals, CBF–FCS coupling was significantly lower in CSM patients. The decrease in CBF–FCS coupling in CSM patients was observed in the superior frontal gyrus, bilateral thalamus, and right calcarine cortex, whereas the increase in CBF–FCS coupling was observed in the middle frontal gyrus. Moreover, low CBF and high FCS were observed in sensorimotor cortices and visual cortices, respectively.

**Conclusion:** In general, neurovascular decoupling at cortical level may be a potential neuropathological mechanism of CSM.

## Introduction

Cervical spondylotic myelopathy (CSM) is a common degenerative spinal cord complication that results in significant mortalities (1–3). Lifestyle and technological advancements changes have paralleled the incidences of CSM (2, 4). Increasing evidence shows that CSM is linked to the altered structure (5) and function (6–11) of the brain. Functional MRI (fMRI), a blood-oxygen-level-dependent signal, measures the spontaneous neural activity of the brain. Assessment of functional alterations in CSM patients relies on regional homogeneity (ReHo), amplitude of low frequency fluctuation (ALFF), and functional connectivity (FC). These methods have revealed extensive functional reorganizations including regional alterations of specific brain regions (e.g., S1, M1, V1) (7, 12–14), circuits (e.g., the thalamus-cortical circuit) (15, 16), subnetworks (e.g., the sensorimotor network and default mode network) (8, 17–19), and the whole brain network (8) in CSM patients. Among the aforementioned neuroimaging metrics based on fMRI technique, FC is the most widely used for exploring brain functional reorganization in CSM patients. The inherent simplicity, sensitivity, and ease of interpretation favors the use of seed-based resting-state FC (20). However, this metric is based on the temporal correlation between time-courses extracted from priori-defined seed-regions; thus, it may be unsuitable if the disease pathology is unknown (21). Whole-brain functional connectivity strength (FCS) analysis is another data-driven method, which considers the temporal correlation between each voxel's time-course and all other voxels' time-courses in the brain (22). FCS is referred to as the voxel-level “degree centrality” in graph theory, and brain regions with high FCS are usually considered functional hubs, intricately and functionally connected with the rest of the brain (21, 23). FCS is applied in investigating connectivity changes in various psychological diseases including schizophrenia, ADHD, and AD, among others. (24–27). Therefore, FCS may also be suitable for investigating cortical functional changes in CSM patients.

Cerebral blood flow (CBF) is the amount of blood delivered to a specific brain tissue over a given time (28–30). Resting-state CBF is strongly related to the brain metabolism (glucose utilization, aerobic glycolysis, and oxygen consumption) (31, 32). Positron emission tomography (PET) and single photon emission computerized tomography (SPECT) have been used in assessing CBF changes in CSM (14). Corresponding assessments show that the spinal cord CBF reflects inflammatory processes in CSM pathology. CBF is also diagnostic and prognostic marker of CSM (33–35). SPECT has revealed significantly low brain rs-CBF in the posterior cortical areas of CSM patients (36). Given that PET and SPECT are invasive procedures, they are cumbersome and have low spatial resolution. Contrarily, the arterial spin labeling (ASL) is a non-invasive MR perfusion technique for measuring rs-CBF using an endogenous contrast agent. Therefore, ASL-based CBF can reveal the metabolic state and inflammatory process in the cortices of CSM patients.

Neurovascular coupling (NVC) is an intrinsic brain function which reflects a strong connection between neuronal activity and blood supply (37). In the brain, the bulk of energy is used in driving spontaneous brain activity (38). Based on neurovascular coupling (NVC) hypothesis, brain regions with higher spontaneous regional neural activity tend to have a greater metabolic demand, which increases blood perfusion (39). Functional hubs in human brain display higher FCS and requires higher metabolic demand (40–42). Numerous network analyses show that CBF reflects both anatomical and functional connections in the brain (FC, ICA, and FCS-based FC) are associated with (43, 44). Across-voxel CBF–FCS correlation (spatial correlation between FCS and CBF in the whole-brain or gray matter voxels) have been used in characterizing the coupling between the neural activity and hemodynamic response in the brain. This approach identifies altered neurovascular coupling in diseases such as schizophrenia, which cannot be detected using FCS or CBF alone (42, 45–47). In recent years, it has been found that altered neurovascular coupling is associated with multiple neuropathology process in the cortex. For instance, abnormal astrocytes or GABA interneurons may disrupt neuronal activity and vascular response (42, 48). Other parameters such as nitric oxide and neuroinflammation levels can also affect neurovascular decoupling.

In the past decades, neuroinflammation, a critical factor affecting neurovascular coupling (49, 50), has been consistently reported in CSM patients (51–54). Herein, we hypothesized that compared with healthy individuals, CSM patients would exhibit a significantly lower CBF–FCS coupling compared with healthy controls. As such, we separately explored FCS and CBF changes in specific brain regions of CSM patients. The resting-state fMRI signals and ASL-based cerebral blood flow analyses in 27 CSM patients, and 24 sex/age-matched healthy participants were collected and analyzed. The spatial correlation between CBF and FCS across voxels were then compared between CSM and healthy individuals. The associations between altered neurovascular coupling and the severity of clinical symptoms in patients with cervical compressive myelopathy based on Japanese Orthopedic Association (JOA) scores was also analyzed. CBF and FCS were also compared between groups.

## Methods

### Participants

The protocol for this study was approved by the ethical review board of Tianjin Hospital, China. All participants consented to participate in this study. In general, 29 CSM patients and 24 healthy individuals were recruited in this study between 2019 and 2020. All CSM patients were right-handed. To be included, CSM patients must have fulfilled the following: (a) with cord compression on cervical spine based on MRI-analysis; (b) with sensorimotor deficit of extremities or bladder/bowel dysfunction; (c) with spinal cord compression; (d) with no history of cervical spine surgery; (e) available for the entire study period; (f) with no stenosis of extracranial vertebral artery and the carotid artery after Doppler ultrasound examination; (g) with no clinical or history of other neurologic, psychiatric, ocular, or systemic diseases like hypertension and diabetes; and (h) with no history of alcohol and substance abuse. The 24 healthy individuals were matched to the CMS patients with regard to age, gender, and education. Specific inclusion criteria included: (a) absence of spine compression, (b) other spinal or brain neurological disorders or systemic disease, and (c) availability for the entire study period. The demographic data of the study participants at baseline are shown in Table 1. Two CSM patients with excessive head motion (more than five times the average scrubbing) were excluded from the study. Therefore, the final analyses were based on 27 CSM patients.

**Table 1:** The demographic data study participants at baseline.

	**CSM**	**HC**	***p*-value**
	**(*n* = 27)**	**(*n* = 24)**	
Age (years)	53.7 ± 8.1	54.2 ± 7.3	0.44
Gender (F/M)	12/14	12/12	0.93
Education (years)	11.7 ± 2.2	11.3 ± 2.1	0.61
Pre-JOA	11.1 ± 1.8		
Post-JOA	15.0 ± 1.2		
JOA recovery	3.92 ± 1.9		
MoCA	23.4 ± 2.6		
MMSE	23.4 ± 1.9		

*Pre, preoperative; Post, postoperative; MoCA, Montreal Cognitive Assessment; MMSE, Mini-Mental State Examination; JOA, Japanese Orthopedics Association scores; JOA-recovery, Preoperative JOA scores minus Postoperative JOA scores*.

### Clinical Assessments

Cognitive aspects of the CSM patients were evaluated using the Montreal Cognitive Assessment (MOCA) and the Mini-Mental State Examination (MMSE). CSM patients underwent a thorough assessment for the severity of cervical compressive myelopathy based on Japanese Orthopedic Association (JOA) scores. Details of clinical assessment for each patient are shown in Supplementary Table 1.

### Acquisition of Clinical Data

The 3T fMRI data were acquired using a MAGNETOM Prisma 3T MR scanner (Siemens, Erlangen, Germany) equipped with a 64-channel phase-array head-neck coil. Participants kept their heads still during the scanning process. The head was supported with a sponge pad to minimize unconscious movement. The participants closed their eyes but remained awake, and restrained from specific and strong thoughts. BOLD signals were captured simultaneously using a prototype multi-slice gradient echo-planar imaging (EPI) sequence. The specific test parameters included 30 ms echo time (TE), 800 ms repetition time (TR), 222 × 222 mm field of view (FOV), 74 × 74 matrix, 3 × 3 mm in-plane resolution, flip angle (FA) of 54 degrees, slice thickness of 3 mm, 0 section thickness, 18 slices, transversal slice orientation, bandwidth of 1,690 Hz/pixel, slice acceleration factor of 4, and phase encoding acceleration factor of 2 under parallel acquisition technique (PAT) mode. Overall, 450 images were captured in 6 min.

A high-resolution 3D T1 structural image (two inversion contrast magnetization prepared rapid gradient echo sequences, MP2RAGE) was also captured under the following parameters: 4,000 ms/3.41 ms TR/TE, inversion time (TI1/TI2) of 700 ms/2,110 ms, FA1/FA2 of 4/5°, 256 × 240 matrix, FOV of 256 × 240 mm, 192 slices, 1 × 1 mm in-plane resolution, 1 mm slice thickness, and sagittal slice orientation. The process lasted 6 min and 42 s. Participants closed their eyes, sat in relaxed position and moved minimally, thought about nothing in particular, and stayed awake during the entire process.

Resting-state perfusion imaging was performed using a pseudo-continuous ASL (pcASL) sequence equipped with a 3D fast spin-echo acquisition and background suppression platform (TR/TE of 3,600/21.8 ms; post-label delay of 2,025 ms; spiral in readout of 8 arms with 512 sample points; FA of 180°; FOV of 220 × 220 mm; reconstruction matrix of 128 × 128; slice thickness of 3 mm, no gap; 44 axial slices; eight excitations; and 284 s of acquisition time). The label and control whole-brain image volumes were eight TRs. In total, eight pairs of label and control volumes were captured.

### fMRI Data Preprocessing

MR data were preprocessed using the toolbox Data Processing Assistant for rs-fMRI (DPARSF) (http://www.restfmri.net/forum/DPARSF) pipeline. Briefly, 450 volumes were acquired for functional scanning. The first 10 volumes of each functional scans were used for acclimatization of scanning and magnetization stabilization and were excluded. Motion correction was performed to remove the influences of head movement (due to significantly shortened TR, slice-timing correction was not applied). Liner-drift, Friston-24 parameters, mean global signal, white matter signal, and CSF signal covariates were regressed out to minimize non-neural signals. Subsequently, a scrubbing step for high motion time-points was also performed. The standard FD Jenkinson value was set at 0.5. Time-points exceeding the threshold were scrubbed using the cubic spine method (scrubbing time-points before bad time-points: 2; scrubbing time-points after bad time-point). Effects of high-frequency noise were removed using a band pass filter (0.01–0.08 Hz). Functional images were co-registered to structural images and spatially normalized using the Montreal Neurological Institute template. Each voxel was resampled to 3 × 3 × 3 mm^3^, and then smoothed with a 6 mm full-width-half-maximum isotropic Gaussian kernel before data analysis.

### CBF Analysis

To quantify CBF, the ASL images were analyzed using SPM12 (Statistical Parametric Mapping) (http://www.fil.ion.ucl.ac.uk/spm/) and ASL Data Processing Toolbox (ASLtbx) (http://www.cfn.upenn.edu). Pulsed ASL data were processed as follows: first, the ASL images were realigned to correct for head motion. Second, the perfusion difference of images was calculated through sinc subtraction of label/control pairs. Third, relative CBF images of each subject were normalized using the SPM12 software based on the standard Montreal Neurological Institute (MNI) template. Finally, spatial smoothing was performed again using an FWHM of 6 mm. The intervening effect of head-motion, education, age and gender was removed using multiple liner regression analysis.

### Functional Connectivity Strength (FCS) Analysis

In this study, we performed voxel-wise FCS analysis. For a given voxel, we (1) first computed Pearson correlation coefficients between the BOLD time-course of this voxel and the rest of voxels within the gray matter mask; (2) the FCS of this voxel is calculated as the number of the coefficients that surpass the threshold of 0.2. In this study, because of the removal of the global signal, there is potential possibility for inducing controversial negative correlations; thus, we restricted our analysis to positive correlations only. Then, this procedure was repeated once for each voxel within the gray matter mask. Therefore, for each subject, we obtained an FCS map and it was spatially smoothed with a 6 × 6 × 6 mm FWHM Gaussian kernel. To remove potential influences of head-motion, education, age, and gender, we set these variables as covariates and regress out these effects by multiple liner regression.

### Whole Gray Matter CBF–FCS Coupling Analysis

For subject-level analysis, both CBF and FCS maps were normalized into z-scores by dividing the SD of global values within the gray matter mask with the difference between the mean and individual global values within the gray matter mask. The coupling between FCS and CBF was based on the spatial correlation between CBF and FCS maps across all voxels within the gray matter mask.

For group-level analysis, the average of both CBF and FCS maps of each group were first obtained. The CBF and FCS maps were then converted into z-scores by subtracting the mean of individual global values and dividing the results by the SD of global values within the gray matter mask. The correlation between CBF and FCS maps of each group were then calculated. To reveal group-level CBF–FCS coupling difference between CSM and HC, the correlation coefficients of the two groups were compared. This analysis was performed using an online platform (http://comparingcorrelations.org) as previously described (55).

Subsequently, the spatial correlation between the CBF and FCS map for each subject was calculated to obtain a subject-level whole-brain CBF–FCS coupling. The difference in subject-level CBF–FCS coupling between CSM and HC was analyzed using a two-sample *t*-test.

### Region-Wise CBF–FCS Coupling Analysis

For region-wise analysis, both CBF and FCS maps were converted into z-scores by subtracting the mean of a group from global values within the gray matter mask and dividing the difference by the corresponding SD of the group. For a given subject, the coupling between FCS and CBF was the spatial correlation between CBF and FCS maps across all voxels within each subregion, based on the Anatomical Automatic Labeling (AAL) template. This generated 116 coefficients representing the CBF–FCS coupling for a given subregion. The difference in CBF and FCS between CSM patients and healthy individuals for a subregion were analyzed using two sample *t*-tests. The resultant *p*-values were corrected by the False Discovery Rate (FDR) method.

### Region-Wise CBF and FCS Analysis

The CBF and FCS maps were first normalized into *z*-scores, then the mean values for each sub-region defined by AAL template were obtained and compared between the two groups. The resultant *p*-values were corrected by FDR for multiple comparison correction.

### Voxel-Wise Comparisons in CBF and FCS

To better explore the differences between CSM patients and healthy individuals, we compared the differences in CBF and FCS between the two groups in a voxel-wise manner while controlling for head motion, education, age, and sex. Multiple voxel-wise comparisons were also performed by FDR at statistical significance of corrected *p* < 0.05.

### ROI-Wise Analysis

Although the multiple comparison correction method (FDR correction) was used in our previous region-wise analyses, this method may overlook important differences within brain regions. Therefore, to better explore functional or structural alterations in CSM patients, 12 regions of interest (ROIs) were selected for further analysis. They included the left and right precentral gyrus, the left and right postcentral gyrus, the left and right supplementary motor area, the left and right calcarine gyrus, the left and right precuneus, and the left and right thalamus (7, 12, 19, 56, 57). The ROIs were described based on the corresponding brain areas defined in the AAL. The association between CBF and FCS between voxels in a given region was also analyzed. The mean CBF and FCS between CSM patients and healthy individuals along age, gender, education years, and head motion were also compared.

### The Relationship Between Altered Brain Function and Specific Clinical Parameters

The possible relationship between the whole-brain level CBF and FCS in the whole gray matter voxels was analyzed using Cox regression analysis. The relationship between CBF as well as FCS and several clinical parameters including preoperative/postoperative JOA scores, JOA recovery, as well as MoCA and MMSE scores was also analyzed. The relationship between CBF as well as FCS and the aforementioned clinical parameters in a given brain region was also evaluated. Finally, the relationship between CBF as well as FCS in ROI and the afore-mentioned clinical parameters was also performed.

## Results

### Whole Gray Matter CBF–FCS Coupling Changes in CSM

**Figure 1:**
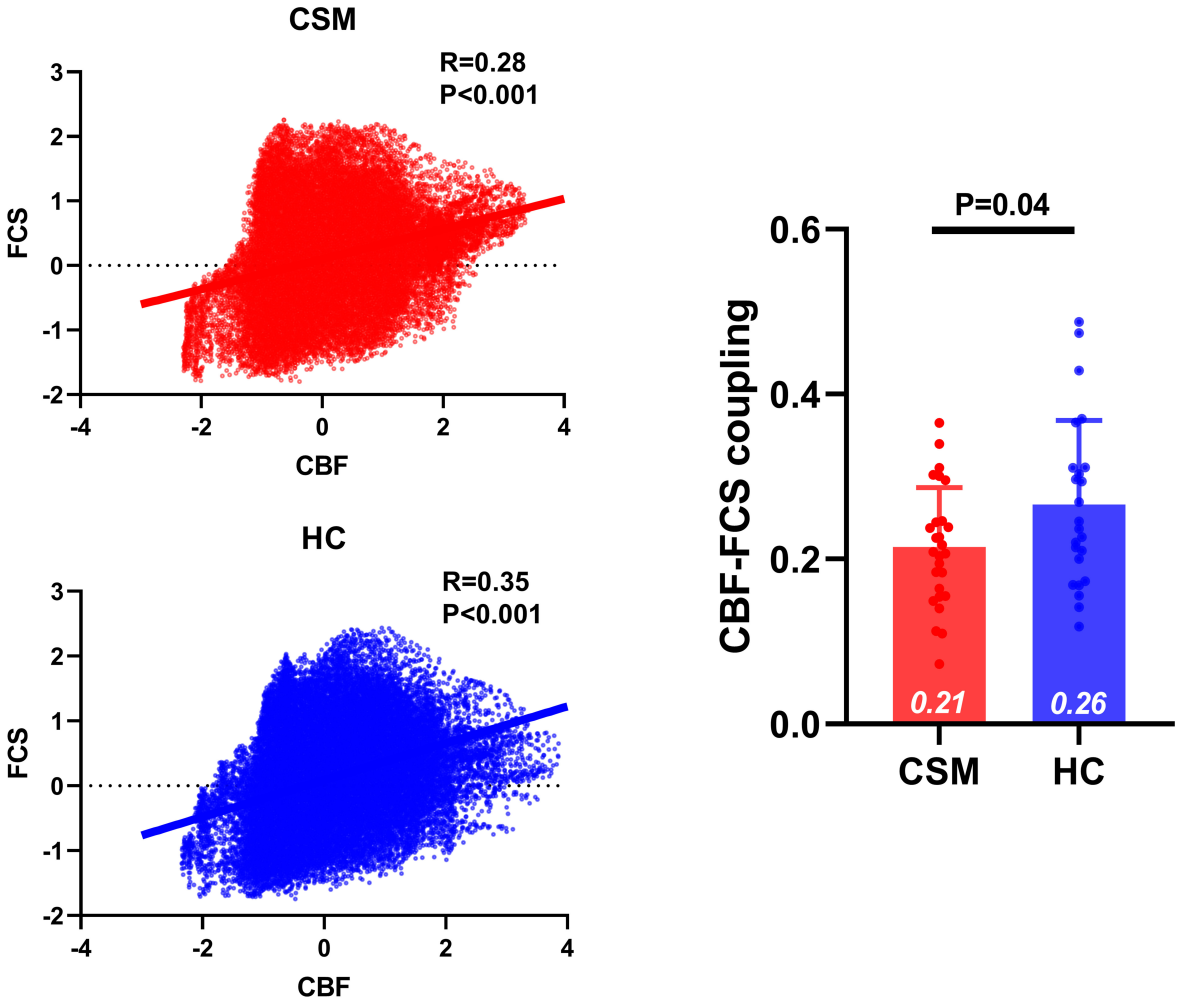
The CBF–FCS coupling changes in the whole gray matter of cervical spondylotic myelopathy (CSM) patients. Scatter plots show the spatial across voxels correlations between CBF and FCS in CSM patients (red) and healthy controls (blue). Although there is significant correlation between CBF and FCS in both CSM patients and healthy individuals, compared to healthy individuals CSM patients display significantly weak CBF–FCS coupling. Error bars represent the SD. CBF, cerebral blood flow; FCS, functional connectivity strength.

Compared with healthy controls, CSM patients exhibited significant decreased CBF–FCS coupling for both subject- and group-level analysis. In group-level analysis, the mean CBF and FCS of each group (i.e., mean maps across subjects within each group) were obtained. The correlation coefficient between group-mean FCS map and group-mean CBF map in CSM was 0.28; the correlation coefficient between group-mean FCS map and group-mean CBF map in HC was 0.33. These two correlation coefficients were compared, and the resultant *z*-value was −46.0489, *p* < 0.0001 [Dunn and Clark's method (58)], *z*-value was −45.6988, *p* < 0.0001 [Steiger's method (59)], and z-value was −46.0489, *p* < 0.0001 [Raghunathan's method (60)]. In subject-level analysis, the correlation coefficients between the CBF map and FCS map across whole gray matter voxels were compared between CSM patients and healthy controls. We observed significantly decreased CBF–FCS coupling in CSM patients (*t*-value = 2.109, *df* = 49, *p* = 0.0401) (Figure 1).

### Region-Wise CBF–FCS Coupling, CBF and FCS Changes in CSM Patients

**Figure 2:**
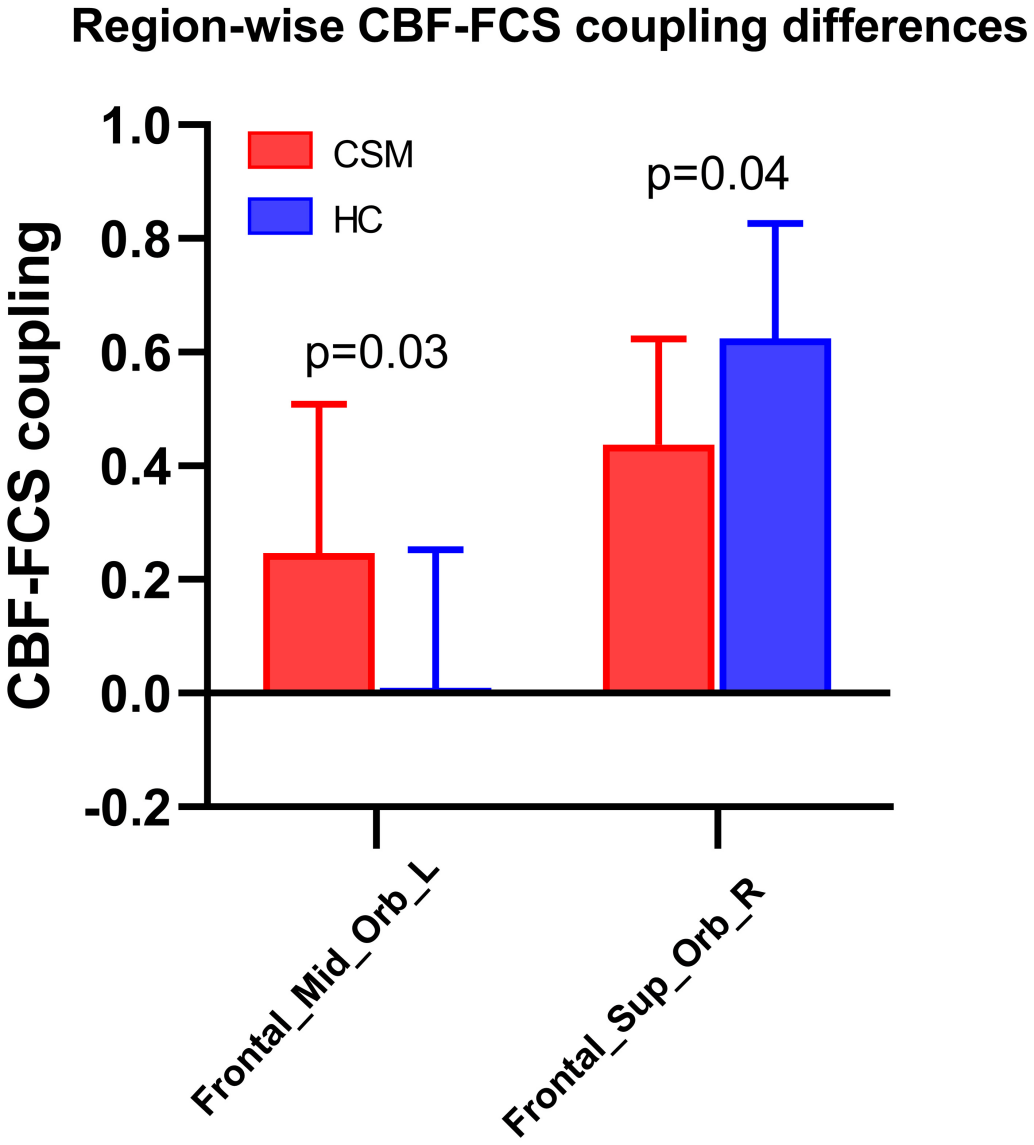
Region-wise CBF–FCS coupling changes based on anatomical automatic labeling (AAL) in cervical spondylotic myelopathy (CSM) patients. Significant CBF-FCS correlation across voxels within the middle frontal gyrus and superior frontal gyrus were observed between CSM patients and healthy individuals. Error bars represent the SD. FDR-corrected *p*-values are shown above the bars. CBF, cerebral blood flow; FCS, functional connectivity strength.

Compared with healthy controls, CSM patients exhibited significantly low CBF–FCS coupling in the superior frontal gyrus (*t*-value = −3.51, q-value = 0.04, FDR corrected), but substantially high CBF–FCS coupling in the middle frontal gyrus (*t*-value = 3.56, *q*-value = 0.03, FDR corrected) (Figure 2).

**Figure 3:**
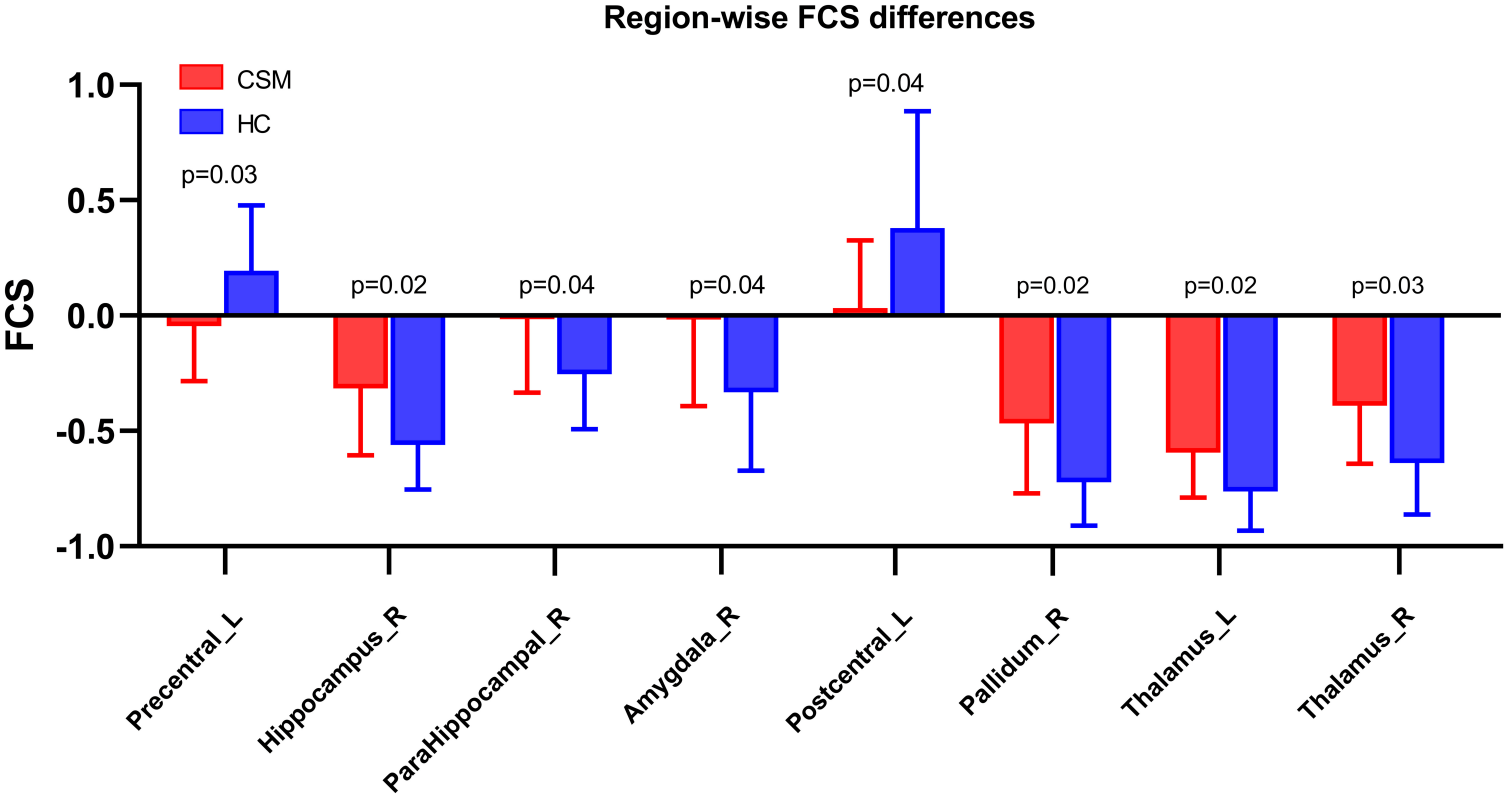
Region-wise CBF–FCS coupling changes in cervical spondylotic myelopathy (CSM) patients based on AAL. FCS was significantly different between CSM patients and healthy individuals. Error bars represent the SD, FDR-corrected *p*-values are shown above bars. CBF, cerebral blood flow; FCS, functional connectivity strength; Precentral_L, Left precentral gyrus; Hippocampus_R, Right hippocampus; ParaHippocampal_R, Right parahippocampus; Amygdala_R, Right Amygdala; Postcentral_L, Left postcentral gyrus; Pallidum_R, Right pallidum; Thalamus_L, Left Thalamus; Thalamus_R, Right thalamus.

Compared with healthy controls, the FCS of CSM patients was significantly low in the left precentral gyrus (*t*-value = −3.54, *q*-value = 0.03, FDR corrected) and left postcentral gyrus (*t*-value = −3.50, *q*-value = 0.04, FDR corrected). Also, the FCS of CSM patients was substantially high in the right hippocampus (*t*-value = 3.62, *q*-value = 0.02, FDR corrected), right parahippocampus (*t*-value = 3.51, *q*-value = 0.04, FDR corrected), right amygdala (*t*-value = 3.49, *q*-value = 0.04, FDR corrected), right pallidum (*t*-value = 3.61, *q*-value = 0.02, FDR corrected), right thalamus (*t*-value = 3.55, *q*-value = 0.03, FDR corrected), and left thalamus (*t*-value = 3.63, *q*-value = 0.02, FDR corrected) (Figure 3).

However, there were no significant differences in region-wise CBF between CSM patients and healthy individuals, possibility because of the small sample size used in this study and the relatively high strict multiple comparison correction method used.

### Voxel-Wise CBF and FCS Changes in CSM Patients

**Figure 4:**
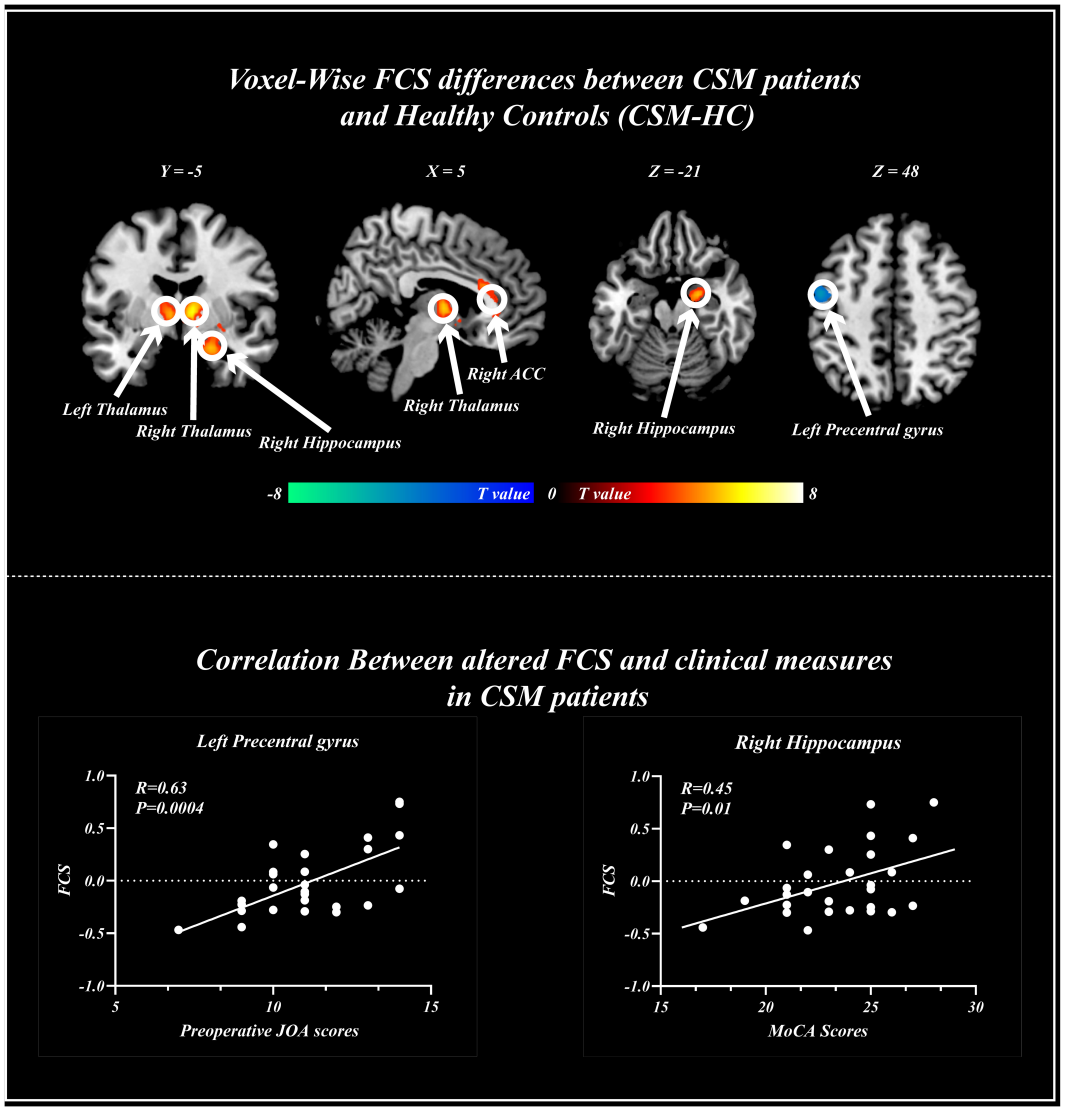
Voxel-wise functional connectivity strength (FCS) changes in cervical spondylotic myelopathy (CSM) patients and healthy patients. Significant difference in FCS was found between the two groups. Scatter plots for the relationship between altered FCS and clinical measures including Montreal cognitive assessments (right), and preoperative Japanese Orthopedics Association scores (Left) across voxels. FCS, functional connectivity strength; ACC, anterior cingulate gyrus.

Compared with healthy individuals, the FCS of CSM patients was significantly high in the left thalamus, right thalamus, right anterior cingulate gyrus, and right hippocampus. Contrarily, the FCS of CSM patients was significantly low in the left postcentral gyrus. Details of these clusters are shown in Table 2. The spatial distribution of the clusters is shown in Figure 4.

**Table 2:** Difference in voxel-wise FCS between CSM patients and healthy individuals.

**Brain regions**	**Peak MNI coordinate**			***t*-value**	**Cluster-size**
	**(x, y, z)**				
**CSM** **>** **HC**					
Left thalamus	9	−3	3	6.31	167
Right thalamus					
Right hippocampus	21	−6	−21	5.43	80
Right ACC	3	24	21	4.81	51
**CSM** **<** **HC**					
Left precentral	−51	−6	48	−4.85	50

*CSM, Cervical Spondylotic Myelopathy; HC, Healthy Controls; ACC, Anterior Cingulate Gyrus*.

### ROI-Wise CBF–FCS Coupling, CBF and FCS Changes in CSM Patients

Voxel-wise and region-wise analysis is highly robust and stringent, given the multiple comparison corrections. Therefore, based on functional alterations in CSM patients, several analyses in ROIs were performed.

**Figure 5:**
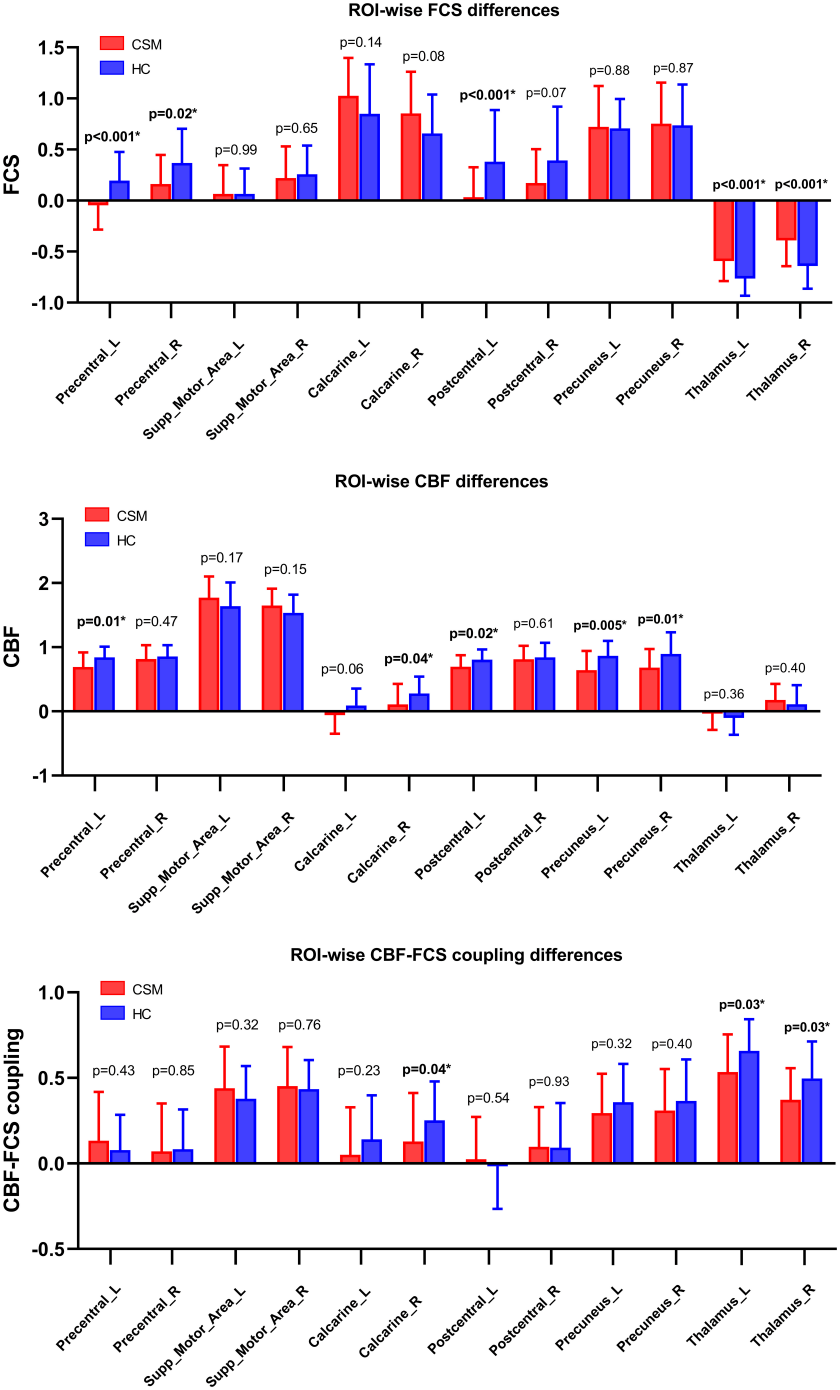
CBF–FCS coupling changes in ROI in cervical spondylotic myelopathy (CSM) patients based on anatomical automatic labeling. We found significant difference in CBF–FCS coupling, as well as CBF rate and FCS. Error bars represent the SD. *P*-values (uncorrected) are shown above bars. CBF, cerebral blood flow; FCS, functional connectivity strength; ROI, region of interests; Precentral_L, Left precentral gyrus; Precentral_R, Right precentral gyrus; Supp_Motor_Area_L, Left supplementary motor area; Supp_Motor_Area_R, Right supplementary motor area; Calcarine_L, Left calcarine cortex; Calcarine_R, Right calcarine cortex; Postcentral_L, Left postcentral gyrus; Postcentral_R, Right postcentral gyrus; Precuneus_L, Left precuneus cortex; Precuneus_R, Right precuneus cortex; Thalamus_L, Left Thalamus; Thalamus_R, Right thalamus.

Compared with healthy individuals, the CBF of CSM patients was significantly low in the left precentral gyrus (*t*-value = −2.65, *p* = 0.01, uncorrected), right calcarine gyrus (*t*-value = −2.05, *p* = 0.04, uncorrected), left postcentral gyrus (*t*-value = −2.27, *p* = 0.02, uncorrected), left precuneus (*t*-value = −2.93, *p* = 0.005, uncorrected), and right precuneus (*t*-value = −2.47, *p* = 0.01, uncorrected) (Figure 5).

The FCS of CSM patients was also significantly low in the left precentral gyrus (*t*-value = −3.54, *p* < 0.001, uncorrected), right precentral gyrus (*t*-value = −2.35, *p* = 0.02, uncorrected), and left postcentral gyrus (*t*-value = −3.41, *p* < 0.001, uncorrected) but substantially high in the left (*t*-value = 3.63, *p* < 0.001, uncorrected), and right thalamus (*t*-value = 3.55, *p* < 0.001, uncorrected) (Figure 5).

CBF–FCS coupling was also low in the right calcarine gyrus (*t*-value = −2.04, *p* = 0.04, uncorrected), left thalamus (*t*-value = −2.20, *p* = 0.03, uncorrected), and right thalamus (*t*-value = −2.23, *p* = 0.03, uncorrected) (Figure 5).

### Correlation Between Brain Alterations and Clinical Measures

Subject-level analysis revealed no correlation between CBF and FCS levels in the whole gray matter of CSM patients (Supplementary Table 2).

Region-wise analysis revealed a strong positive correlation between CBF as well as FCS in the left middle frontal gyrus and postoperative JOA scores (severity of clinical symptoms in patients with cervical compressive myelopathy) in CSM patients (*r* = 0.50, *p* < 0.05, uncorrected) (Supplementary Table 3). The FCS in the left precentral gyrus also positively correlated with preoperative JOA scores (*r* = 0.53, *p* < 0.05) (Supplementary Table 4).

Voxel-wise analysis revealed that the FCS in the left precentral gyrus positively correlated with the preoperative JOA scores (*r* = 0.63, *p* < 0.001), whereas the FCS in the right hippocampus positively correlated with MoCA scores (*r* = 0.45, *p* < 0.05) (Figure 4).

ROI-wise analysis on its part revealed a strong negative correlation between postoperative JOA scores and CBF as well as FCS and in the left calcarine gyrus (*r* = −0.37, *p* < 0.05). A negative correlation was also observed between preoperative JOA scores and CBF as well as FCS in the right calcarine gyrus (*r* = −0.37, *p* < 0.05). A strong negative correlation was observed between preoperative JOA scores and CBF as well as FCS in the left thalamus (*r* = −0.48, *p* < 0.05). We also found a negative correlation between postoperative JOA scores and CBF as well as FCS in the right thalamus (*r* = −0.38, *p* < 0.05) (Supplementary Table 5). Preoperative JOA score positively correlated with FCS in the left precentral gyrus. We observed a negative correlation between the FCS in the left calcarine and preoperative JOA scores (*r* = −0.37, *p* < 0.05) as well as JOA recovery (*r* = 0.40, *p* < 0.05) following decompression surgery. The FCS in the left precuneus negatively correlated with preoperative JOA scores (*r* = −0.54, *p* < 0.05) as well as JOA recovery (*r* = 0.50, *p* < 0.05), whereas the FCS in the right precuneus negatively correlated with preoperative JOA scores (*r* = −0.61, *p* < 0.05) as well as JOA recovery (*r* = 0.53, *p* < 0.05) (Supplementary Table 6). In this section, no multiple comparison correction method was performed because the relative strict multiple comparison method may cover up the mild association between brain variables and clinical measures within the selected ROIs.

## Discussion

To the best of our knowledge, this is the first study investigating CBF–FCS coupling changes in CSM patients using a combination of BOLD and ASL techniques. We found that, compared with healthy individuals, the CBF–FCS coupling in the whole gray matter was significantly low. CSM patients also exhibited altered CBF–FCS coupling in the superior/mid frontal gyrus. Moreover, the FCS in the pre/postcentral gyrus was substantially low in CSM patients, in contrast with rCBF in the thalamus, hippocampus, and visual cortices, which were all substantially high. These findings deepen our understanding on the neural pathological characteristics of CSM. We used blood oxygenation level-dependent (BOLD) signals in exploring functional alterations in the brain under certain disease complications such as Alzheimer's disease (AD), Parkinson's disease (PD), epilepsy, attention deficit hyperactivity disorder (ADHD), and mood disorders.

We observed a significant whole-brain wise across-voxel correlation between rCBF and FCS in healthy adults, consistent with previous findings (27, 45). Proper neurovascular functioning is necessary for normal coupling between rCBF and FCS (both neuronal and vascular components). Astrocytes maintain normal coupling between neuronal and vascular components by coordinating the activity and responses mediated by these components (48). Although we observed a significant across-voxel correlation between rCBF and FCS in CSM patients, it was substantially lower than that of healthy patients, implying decoupling between rCBF and FCS in CSM patients. This neurovascular decoupling may have resulted from prolonged neuroinflammation caused by myelopathy (61–63), in which the abnormally functioning astrocytes in CSM patients may have reduced the regional neural activity and blood supply (62, 64, 65). Neuroinflammation would have also directly affected the vascular component, leading to neurovascular decoupling in CSM patients. However, we did not find a significant difference in rCBF between CSM patients and healthy individuals. Therefore, neurovascular decoupling in CSM is caused by abnormal functioning of the astrocyte.

We observed low across-voxel correlation between rCBF and FCS in the superior frontal gyrus of CSM patients. The superior frontal gyrus regulates motor functions. Previous neuroimaging studies have demonstrated that CSM patients display altered neural activity in the superior frontal gyrus. Takenaka et al. observed significantly high functional connectivity between superior frontal and lingue gyrus in (healthy individuals), and surgery substantially improved this phenomenon in CSM patients (66). In their subsequent study using the same cohort, Takenaka et al. found that decompression surgery increased ALFF in the superior frontal gyrus of CSM patients (9). Herein, we found a high across-voxel correlation between rCBF and FCS in the middle frontal gyrus of (CSM patients), a brain region that participates in processing chronic pain (67, 68). The high neurovascular coupling may have resulted from the chronic pain in CSM patients. Taken together, CSM patients display altered superior frontal and middle frontal gyrus function. Meanwhile, the neurovascular coupling (measured by cross-voxel correlation between rCBF and FCS) provides complementary information on the pathological changes in CSM.

FCS levels reflect the contribution of each voxel in information transmission in the whole brain. Information transmission efficiency is directly proportional to HFCS levels. Low FCS implies local brain damage (reduction in gray matter and alterations in white matter) (69, 70). Herein, we found FCS within sensorimotor cortices including pre and postcentral gyrus, and the FCS within left precentral gyrus was significantly low in CSM patients, and positively correlated with preoperative JOA scores. This implies that regional impairments are directly proportional to the severity of clinical symptoms. We speculate the functional impairments result from the structural damage in sensorimotor cortices in CSM patients. Converging evidence shows that CSM patients have little gray matter (71–73) and low metabolism in the sensorimotor cortices (14, 74). Therefore, the structural changes may result from the FCS changes in the precentral and postcentral gyrus. Moreover, we also observed a significant increase in FCS within the thalamus and hippocampus of CSM patients, and high FCS in the hippocampus positively correlated with MoCA scores. High global efficiency of the default mode network positively correlates with MoCA scores in CSM patients. This implies that the high global efficiency in the default mode network compensates for the local impairment (measured by signal variability) in the default mode network (19). As the hippocampus has been shown to be an important brain region participating in many cognitive functions, the high FCS in the hippocampus was also associated with compensatory or adaptive changes in CSM, necessary for maintaining normal cognitive function in these individuals.

What causes functional changes in the brain of CSM patients is largely unknown. However, it is thought that CSM patients develop stenosis of transverse foramen that compresses vertebral artery, altering cortical blood supply. However, in our current study, altered CBF in CSM patients was only observed in a few regions. We found no significant voxel-wise or region-wise difference in CBF between CSM patients and healthy individuals. However, we observed altered CBF and FCS coupling in CSM patients, consistent with the idea that functional impairments in CSM patients is caused by neuroinflammation.

Regarding limitations, first, the CSM patients included in our study received a long conservative treatment before surgery (NSAID), which could have influenced the observed outcomes. Future studies using treatment-naïve CSM patients are necessary to validate our findings. Second, both CBF and FCS are indirect measures of vascular and neuronal function; thus, our findings may not be conclusive. Third, postoperative fMRI assessment was not performed due to the possible artifacts of the surgical implants and possible heating of these materials. Means of conducting safe postoperative fMRI are necessary to allow this process in patients with surgical implants. Lastly, the sample size of our current study is relatively small. Future studies with a larger sample size are needed to further confirm our conclusion.

In conclusion, we observed an altered neurovascular coupling in CSM by combining BOLD and ASL techniques. Specifically, we found a decreased across-voxel correlation between rCBF and FCS in the superior frontal gyrus involved in motor control and motor planning, and an increased across-voxel correlation between rCBF and FCS in the middle frontal gyrus implicated in cognitive function. These findings suggest that the neurovascular decoupling in the brain may be a potential neural mechanism involved in the pathophysiology of CSM.

## Data Availability Statement

The original contributions presented in the study are included in the article/Supplementary Material, further inquiries can be directed to the corresponding author/s.

## Ethics Statement

The studies involving human participants were reviewed and approved by TianJin Hospital. The patients/participants provided their written informed consent to participate in this study.

## Author Contributions

JL designed the study. WW wrote the manuscript. TW, BL, and TA collected the data. All authors contributed to the article and approved the submitted version.

## Funding

This work was supported by the Tianjin Health Commission Foundation (Grant Number: ZC20192), Tianjin Biomedical Engineering Technology Key Project fund (Grant Number: 18ZXSGSY00010), Tianjin Science Technology Key Project fund (Grant Number: 18YFZCSY00890), and Natural Science Foundation of Tianjin fund (Grant Number: 20JCQNJC01170).

## Conflict of Interest

The authors declare that the research was conducted in the absence of any commercial or financial relationships that could be construed as a potential conflict of interest.

## Publisher's Note

All claims expressed in this article are solely those of the authors and do not necessarily represent those of their affiliated organizations, or those of the publisher, the editors and the reviewers. Any product that may be evaluated in this article, or claim that may be made by its manufacturer, is not guaranteed or endorsed by the publisher.
