# Supplementary material for: Altered Coupling Between Resting-State Cerebral Blood Flow and Functional Connectivity Strength in Cervical Spondylotic Myelopathy Patients
Source: Front Neurol. 2021 Sep 8;12:713520. doi: 10.3389/fneur.2021.713520 (PMC8455933; doi:10.3389/fneur.2021.713520)
Supplement: Supplementary file 1 [file Data_Sheet_1.docx]

***S-Table 1. Behavior scale scores of each patient***

| Patients | Pre-JOA | Post-JOA | JOA recovery | MMSE | MoCA |
| --- | --- | --- | --- | --- | --- |
| CSM001 | 7 | 12 | 5 | 22 | 21 |
| CSM002 | 9 | 15 | 6 | 20 | 22 |
| CSM003 | 11 | 14 | 3 | 23 | 25 |
| CSM004 | 10 | 15 | 5 | 24 | 25 |
| CSM005 | 11 | 15 | 4 | 20 | 19 |
| CSM006 | 9 | 14 | 5 | 22 | 21 |
| CSM007 | 12 | 14 | 2 | 22 | 21 |
| CSM008 | 14 | 14 | 0 | 24 | 25 |
| CSM009 | 14 | 17 | 3 | 23 | 25 |
| CSM010 | 10 | 16 | 6 | 20 | 17 |
| CSM011 | 13 | 14 | 1 | 22 | 23 |
| CSM012 | 14 | 14 | 0 | 23 | 24 |
| CSM013 | 11 | 15 | 4 | 24 | 25 |
| CSM014 | 10 | 15 | 5 | 23 | 25 |
| CSM015 | 11 | 16 | 5 | 22 | 23 |
| CSM016 | 11 | 17 | 6 | 26 | 28 |
| CSM017 | 12 | 16 | 4 | 23 | 25 |
| CSM018 | 9 | 15 | 6 | 25 | 23 |
| CSM019 | 9 | 14 | 5 | 23 | 21 |
| CSM020 | 11 | 16 | 4 | 25 | 22 |
| CSM021 | 10 | 17 | 7 | 25 | 24 |
| CSM022 | 13 | 14 | 1 | 24 | 27 |
| CSM023 | 11 | 15 | 4 | 27 | 26 |
| CSM024 | 11 | 15 | 4 | 22 | 21 |
| CSM025 | 10 | 15 | 5 | 25 | 22 |
| CSM026 | 13 | 16 | 3 | 27 | 27 |
| CSM027 | 14 | 16 | 2 | 26 | 26 |

***S-Table 2. The correlation coefficients between the whole grey matter CBF-FCS coupling and behavior scale scores in CSM patients***

|  | MMSE | MoCA | Pre-JOA | Post-JOA | JOA-recovery |
| --- | --- | --- | --- | --- | --- |
| CBF-FCS coupling | 0.11 | 0.17 | 0.23 | 0.07 | -0.13 |

Pre: Preoperative; Post: Postoperative; MoCA: Montreal Cognitive Assessment; MMSE: Mini-Mental State Examination; JOA: Japanese Orthopedics Association scores; JOA-recovery: Preoperative JOA scores minus Postoperative JOA scores.

***S-Table 3. The correlation coefficients between the behavior scale scores and the altered Region-wise CBF-FCS coupling in CSM patients.***

|  | MMSE | MoCA | Pre-JOA | Post-JOA | JOA-recovery |
| --- | --- | --- | --- | --- | --- |
| L middle frontal gyrus | 0.10 | 0.12 | 0.27 | **0.50*** | 0.03 |
| R superior frontal gyrus | -0.11 | -0.05 | 0.26 | 0.20 | -0.13 |

Pre: Preoperative; Post: Postoperative; MoCA: Montreal Cognitive Assessment; MMSE: Mini-Mental State Examination; JOA: Japanese Orthopedics Association scores; JOA-recovery: Preoperative JOA scores minus Postoperative JOA scores; *: P<0.05

***S-Table 4. The correlation coefficients between the behavior scale scores and the altered Region-wise FCS in CSM patients.***

|  | MMSE | MoCA | Pre-JOA | Post-JOA | JOA-recovery |
| --- | --- | --- | --- | --- | --- |
| L precentral gyrus | 0.18 | 0.23 | **0.53*** | 0.04 | 0.05 |
| R hippocampus | 0.21 | 0.01 | -0.17 | 0.05 | 0.19 |
| R para hippocampus | 0.11 | -0.07 | -0.05 | 0.04 | 0.07 |
| R amygdala | 0.33 | 0.27 | 0.08 | 0.01 | 0.07 |
| L postcentral gyrus | 0.15 | 0.28 | 0.06 | -0.17 | -0.17 |
| R pallidum | 0.30 | 0.06 | -0.12 | -0.06 | 0.08 |
| L thalamus | 0.06 | -0.13 | -0.20 | -0.19 | 0.32 |
| R thalamus | 0.12 | 0.11 | 0.17 | 0.27 | 0.34 |

Pre: Preoperative; Post: Postoperative; MoCA: Montreal Cognitive Assessment; MMSE: Mini-Mental State Examination; JOA: Japanese Orthopedics Association scores; JOA-recovery: Preoperative JOA scores minus Postoperative JOA scores; *: P<0.05

***S-Table 5. The correlation coefficients between the behavior scale scores and the altered ROI-wise FCS-CBF coupling in CSM patients.***

|  | MMSE | MoCA | Pre-JOA | Post-JOA | JOA-recovery |
| --- | --- | --- | --- | --- | --- |
| L precentral gyrus | -0.12 | 0.11 | 0.01 | 0.16 | 0.09 |
| R precentral gyrus | -0.25 | -0.17 | 0.02 | -0.08 | -0.06 |
| L SMA | 0.20 | 0.21 | 0.05 | 0.21 | 0.07 |
| R SMA | 0.11 | 0.21 | 0.25 | 0.34 | -0.04 |
| L calcarine | 0.08 | -0.16 | -0.18 | **-0.37*** | -0.05 |
| R calcarine | 0.10 | -0.12 | **-0.37*** | -0.14 | 0.27 |
| L postcentral gyrus | -0.22 | 0.04 | -0.07 | 0.07 | 0.11 |
| R postcentral gyrus | -0.22 | -0.12 | -0.03 | 0.10 | 0.09 |
| L precuneus | -0.04 | 0.26 | 0.15 | -0.16 | -0.24 |
| R precuneus | 0.15 | 0.21 | 0.32 | 0.18 | -0.20 |
| L thalamus | -0.11 | -0.18 | **-0.48*** | -0.24 | 0.31 |
| R thalamus | -0.12 | -0.05 | -0.21 | **-0.38*** | -0.14 |

Pre: Preoperative; Post: Postoperative; MoCA: Montreal Cognitive Assessment; MMSE: Mini-Mental State Examination; JOA: Japanese Orthopedics Association scores; JOA-recovery: Preoperative JOA scores minus Postoperative JOA scores; *: P<0.05

***S-Table 6. The correlation coefficients between the behavior scale scores and the altered ROI-wise FCS in CSM patients.***

|  | MMSE | MoCA | Pre-JOA | Post-JOA | JOA-recovery |
| --- | --- | --- | --- | --- | --- |
| L precentral gyrus | 0.18 | 0.23 | **0.53** | 0.04 | 0.05 |
| R precentral gyrus | 0.17 | 0.25 | 0.27 | 0.14 | 0.08 |
| L SMA | 0.27 | 0.29 | 0.01 | 0.03 | 0.01 |
| R SMA | 0.24 | 0.30 | 0.11 | 0.01 | 0.09 |
| L calcarine | -0.17 | -0.26 | **-0.37*** | 0.06 | **0.40*** |
| R calcarine | -0.14 | -0.14 | -0.29 | 0.11 | 0.35 |
| L postcentral gyrus | 0.15 | 0.28 | 0.06 | -0.17 | -0.17 |
| R postcentral gyrus | 0.23 | 0.21 | 0.08 | 0.11 | 0.15 |
| L precuneus | -0.03 | -0.13 | **-0.54*** | -0.03 | **0.50*** |
| R precuneus | -0.08 | -0.16 | **-0.61*** | -0.10 | **0.53*** |
| L thalamus | 0.06 | 0.14 | -0.20 | 0.19 | 0.31 |
| R thalamus | 0.12 | 0.11 | -0.17 | 0.27 | 0.33 |

Pre: Preoperative; Post: Postoperative; MoCA: Montreal Cognitive Assessment; MMSE: Mini-Mental State Examination; JOA: Japanese Orthopedics Association scores; JOA-recovery: Preoperative JOA scores minus Postoperative JOA scores; *: P<0.05

***S-Table 7. The correlation coefficients between the behavior scale scores and the altered ROI-wise CBF in CSM patients.***

|  | MMSE | MoCA | Pre-JOA | Post-JOA | JOA-recovery |
| --- | --- | --- | --- | --- | --- |
| L precentral gyrus | 0.25 | 0.15 | -0.12 | 0.15 | 0.22 |
| R precentral gyrus | 0.14 | 0.16 | -0.27 | -0.07 | 0.22 |
| L SMA | -0.15 | 0.13 | 0.02 | -0.25 | -0.17 |
| R SMA | 0.04 | 0.22 | 0.23 | 0.04 | -0.20 |
| L calcarine | 0.10 | 0.07 | 0.04 | -0.06 | -0.08 |
| R calcarine | 0.22 | 0.14 | -0.10 | 0.10 | 0.15 |
| L postcentral gyrus | 0.21 | 0.26 | 0.20 | 0.24 | -0.04 |
| R postcentral gyrus | 0.02 | 0.14 | -0.14 | -0.25 | -0.01 |
| L precuneus | -0.21 | -0.24 | -0.15 | 0.03 | 0.16 |
| R precuneus | -0.07 | -0.08 | -0.23 | -0.15 | 0.12 |
| L thalamus | 0.17 | -0.22 | 0.03 | 0.23 | 0.10 |
| R thalamus | -0.16 | -0.07 | 0.09 | 0.27 | 0.07 |

Pre: Preoperative; Post: Postoperative; MoCA: Montreal Cognitive Assessment; MMSE: Mini-Mental State Examination; JOA: Japanese Orthopedics Association scores; JOA-recovery: Preoperative JOA scores minus Postoperative JOA scores; *: P<0.05
